# Associations between olfactory pathway gene methylation marks, obesity features and dietary intakes

**DOI:** 10.1186/s12263-019-0635-9

**Published:** 2019-04-25

**Authors:** Omar Ramos-Lopez, Jose I. Riezu-Boj, Fermin I. Milagro, M. Angeles Zulet, Jose L. Santos, J. Alfredo Martinez, MENA project, A. Alonso, C. Arancibia, F. Arós, A. Astrup, N. Babio, V. Blázquez, I. Bondia-Pons, L. Brennan, P. Buil-Cosiales, J. Campión, L. R. Cataldo, C. Celis-Morales, D. Corella, M. I. Covas, S. Dalskov, H. Daniel, A. De Arce, R. de la Iglesia, R. Estruch, J. Fernández-Crehuet, M. Fiol, M. Fitó, M. Flores, L. Forga, J. Galgani, E. R. Gibney, M. J. Gibney, A. M. Gómez-Úriz, P. González-Muniesa, E. Goyenechea, B. Guy-Grand, T. Handjieva-Darlenska, C. Holst, A. E. Huerta, S. Jebb, A. Kafatos, M. Kunesová, R. M. Lamuela-Raventós, D. Langin, J. Lapetra, T. M. Larsen, A. López De Munain, P. López-Legarrea, J. A. Lovegrove, I. Macdonald, Y. Manios, M. T. Martínez-Zabaleta, J. C. Mathers, M. Morales, M. A. Muñoz, P. Olmos, O. Pedersen, M. Petersen, A. Pfeiffer, X. Pintó, F. Pollak, P. L. Prieto-Hontoria, E. Ros, S. Rössner, V. Ruiz-Gutierrez, J. Salas-Salvadó, W. H. Saris, L. Serra-Majem, T. I. Sørensen, J. V. Sorlí, V. Stich, M. A. Taylor, E. Toledo, S. Toubro, I. Traczyk, J. P. Valderas, M. van Baak, J. Vega, C. Verdich, M. Walsh, I. Yévenes, I. Abete, A. B. Crujeiras, M. Cuervo, L. Goni, A. Marti, M. A. Martinez-Gonzalez, M. J. Moreno-Aliaga, S. Navas-Carretero, R. San-Cristobal

**Affiliations:** 1 Department of Nutrition, Food Science and Physiology, and Center for Nutrition Research, University of Navarra, 1 Irunlarrea Street, 31008 Pamplona, Spain; 2 Medical and Psychology School, Autonomous University of Baja California, Tijuana, Baja California Mexico; 3 Navarra Institute for Health Research (IdiSNA), Pamplona, Spain; 4 CIBERobn, Fisiopatología de la Obesidad y la Nutrición; Carlos III Health Institute, Madrid, Spain; 5 Department of Nutrition, Diabetes and Metabolism, School of Medicine, Pontificia Universidad Católica de Chile, Santiago, Chile; 6 Madrid Institute of Advanced Studies (IMDEA Food), Madrid, Spain

**Keywords:** Olfactory system, Smell, Epigenetics, OR2Y1, OR4D2, Diet

## Abstract

**Background:**

Olfaction is an important sense influencing food preferences, appetite, and eating behaviors. This hypothesis-driven study aimed to assess associations between olfactory pathway gene methylation signatures, obesity features, and dietary intakes.

**Methods:**

A nutriepigenomic analysis was conducted in 474 adults from the Methyl Epigenome Network Association (MENA) project. Anthropometric measurements, clinical data, and serum metabolic profiles of the study population were obtained from structured databases of the MENA cohorts. Habitual dietary intake was assessed using a validated semiquantitative food frequency questionnaire. DNA methylation was measured in circulating white blood cells by microarray (Infinium Human Methylation 450 K BeadChips). FDR values (*p* < 0.0001) were used to select those CpGs that showed the best correlation with body mass index (BMI) and waist circumference (WC). Pathway analyses involving the characterization of genes involved in the olfactory transduction system were performed using KEGG and pathDIP reference databases.

**Results:**

Overall, 15 CpG sites at olfactory pathway genes were associated with BMI (*p* < 0.0001) and WC (*p* < 0.0001) after adjustments for potential confounding factors. Together, methylation levels at the15 CpG sites accounted for 22% and 20% of the variability in BMI and WC (*r*^2^ = 0.219, *p* < 0.001, and *r*^2^ = 0.204, *p* < 0.001, respectively). These genes encompassed olfactory receptors (*OR4D2*, *OR51A7*, *OR2T34*, and *OR2Y1*) and several downstream signaling molecules (*SLC8A1*, *ANO2*, *PDE2A*, *CALML3*, *GNG7*, *CALML6*, *PRKG1*, and *CAMK2D*), which significantly regulated odor detection and signal transduction processes within the complete olfactory cascade, as revealed by pathway enrichment analyses (*p* = 1.94 × 10^–10^). Moreover, *OR4D2* and *OR2Y1* gene methylation patterns strongly correlated with daily intakes of total energy (*p* < 0.0001), carbohydrates (*p* < 0.0001), protein (*p* < 0.0001), and fat (*p* < 0.0001).

**Conclusions:**

The results of this study suggest novel relationships between olfactory pathway gene methylation signatures, obesity indices, and dietary intakes.

## Background

Obesity epidemic represents one of the most important health challenges worldwide [1]. It has been estimated that about 1.9 billion adults present overweight or obesity based on body mass index (BMI), with an overall prevalence of 39% [2]. Epidemiological studies have identified high BMI as a risk factor for an expanding set of chronic diseases including cardiovascular disease, diabetes mellitus, non-alcoholic fatty liver disease, and many types of cancer, with relevant negative economic and social impacts [3]. At a global level, excessive weight (BMI ≥ 25) accounted for 4 million deaths (7.2% of all-cause deaths) and 120 million deaths and disability-adjusted life years (4.9% of all-cause DALYs) among adults in 2015 [4].

In general, the overconsumption of energy-dense foods, coupled with the adoption of a sedentary lifestyle, is the main environmental factors contributing to the development of obesity and associated clinical manifestations [5]. Moreover, genetic and epigenetic signatures play a role in determining individual susceptibility to fat accumulation [6, 7]. In the past, and until recently, research on obesity has focused on characterizing the biological factors that predispose people to excessive food intake and associated weight gain [8]. In this context, it has been reported that sensorial modalities, including taste and olfaction [9], may influence food preferences and eating behaviors.

In particular, olfactory ability is related to perceived food palatability [10] and may be modulated by hunger/satiety states [11, 12]. Also, exposure to food odors increases appetite for products with similar characteristics in terms of taste and energy density [13]. Indeed, a link between dietary intake and olfactory sensitivity to fat was found [14], although another study did not find changes in energy intake or food preferences in response to odors exposure in women [15]. In addition, fluctuations in the internal levels of carbohydrates, amino acids, and fats could modulate olfactory sensitivity to adjust feeding behaviors in order to maintain nutrient homeostasis [16].

Odor perception during eating depends on the interaction between olfactory and gustatory systems [17]. Besides physiological issues (i.e., age, circadian rhythmicity, endocrine secretions), sensory variability can be also driven by genetic and epigenetic marks, which in turn can impact food consumption and subsequent health outcomes [18]. Associations between taste receptors polymorphisms, dietary intakes, lipid disorders, and liver disease in Mexican subjects were reported [19–22]. Likewise, sequence variants in olfactory receptor genes appeared to contribute to the predisposition to extreme obesity [23, 24] and influenced eating behaviors and adiposity levels [25]. Additionally, DNA methylation patterns at sweet taste pathway genes were associated with BMI and carbohydrate intake in an adult population [26]. Together, these findings suggest the involvement of sensory factors, including olfaction, in appetite regulation and obesity predisposition, where epigenetics may play a crucial role. This hypothesis-driven study aimed to assess associations between olfactory pathway gene methylation signatures, obesity features, and dietary intakes.

## Materials and methods

### Subjects

A nutriepigenomic analysis was conducted in an adult population from the Methyl Epigenome Network Association (MENA) project (*n* = 474), which is constituted by previous clinical trials [27–34]. The study protocol, data confidentiality, and research procedures were in accordance with the ethical principles on human experimentation stipulated in the 2013 Declaration of Helsinki by the World Medical Association [35].

### Study variables

Anthropometric measurements, clinical data, and serum metabolic profiles of the study population were obtained from structured databases of the MENA cohorts. These variables included weight, height, waist circumference (WC), systolic blood pressure (SBP), diastolic blood pressure (DBP), glucose, insulin, total cholesterol, high-density lipoprotein cholesterol (HDL-c), low-density lipoprotein cholesterol (LDL-c), and triglycerides. BMI was calculated as the ratio between weight (kg) and height (m^2^). Insulin resistance was estimated by the homeostatic model assessment-insulin resistance (HOMA-IR) index using the following formula: HOMA-IR = fasting insulin (mU/L) X plasma glucose (mmol/L)/22.5. Triglyceride-glucose (TyG) index was calculated as a predictor of diabetes, as described elsewhere [36].

### Dietary assessment

Dietary data was available from 247 subjects of the PREDIMED, RESMENA, and OBEKIT cohorts, which presented similar characteristics regarding the whole sample. A validated semiquantitative food frequency questionnaire [37] was used to assess the habitual consumption of 137 food items during the previous year according to four frequency categories (daily, weekly, monthly, or never). The obtained food portions and serving sizes were further converted to daily energy (kcal) and macronutrient intakes (g) using the Spanish food composition tables [38].

### DNA methylation analyses

Venous blood samples were drawn by venipuncture after a 12-h overnight fast. White blood cells (WBC) were separated from whole blood by centrifugation at 3500 rpm, at 4 °C for 15 min, and immediately frozen at − 80 °C in buffy coat until assay, as described elsewhere [39]. Genomic DNA was isolated from WBC with the Master Pure kit (Epicentre Biotechnologies, Madison, WI, USA). Purified DNA was quantified by the PicoGreen® dsDNA Quantitation Reagent (Invitrogen, Carlsbad, CA, USA). High-quality DNA samples were modified with sodium bisulfite by using the EZ-96 DNA Methylation kit (Zymo Research Corporation, Irvine, CA, USA) according to the manufacturer’s protocol. Bisulfite-treated DNA samples were hybridized to Infinium Human Methylation 450 K BeadChips (Illumina, San Diego, CA, USA) and scanned using the Illumina HiScanSQ system. Image intensities were obtained with the GenomeStudio Methylation Software Module, v1.9 (Illumina, San Diego, CA, USA). DNA methylation data pre-processing has been recently described elsewhere [40]. Briefly, CpG methylation levels were expressed as β values ranging from 0 (unmethylated) to 1 (methylated) [41], which were corrected for type I and type II bias applying the peak-based correction. Data were normalized in R using a categorical Subset Quantile Normalization method [42]. Probes containing single-nucleotide polymorphisms, hybridizing to multiple genomic locations, or associated with X and Y chromosomes were removed from the analysis. Furthermore, DNA methylation variation explained due to different cell subtypes was corrected following the Houseman procedure [43].

### Pathway analyses

Pathway mapping of genes involved in olfactory transduction (map04740) was performed using the Kyoto Encyclopedia of Genes and Genomes (KEGG) reference database (http://www.genome.jp/kegg/pathway.html). Subsequently, pathway enrichment analyses (confidence level of 99%) were further run in the Pathway Data Integration Portal (pathDIP) platform, University of Toronto, Canada (http://ophid.utoronto.ca/pathdip/).

### Statistical analyses

Data normality was screened by the Kolmogorov-Smirnov test. Principal variables including BMI, WC, energy, and macronutrient intakes were normally distributed (*p* > 0.05). Continuous variables were expressed as means ± standard deviations, while categorical variables were presented as number of cases and percentages. Statistical analyses were performed in the IBM SPSS software, version 20 (IBM Inc., Armonk, NY, USA). A linear regression model (for BMI outcome) was computed in the LIMMA package for R software, which was adjusted by age, sex, study cohorts, and DNA methylation chips. Also, the Benjamini-Hochberg correction for multiple comparisons was applied. Statistically significant thresholds were based on false discovery rate (FDR) cut-offs (*p* < 0.05) and B-statistic values (> 0) for BMI-related analysis. Afterwards, stricter FDR (*p* < 0.0001) were used to select those CpGs that showed the best correlation with BMI. Linear regression analyses adjusted by age and sex were further performed to evaluate correlations of methylation values at olfactory transducing genes with anthropometric (BMI, WC), metabolic (glucose, insulin, lipid profile, HOMA-IR, TyG index), and dietary measurements (total energy and macronutrient intakes). Figure plots showing significant correlations were created in the GraphPad Prism® program, version 6.0C (La Jolla, CA, USA).

## Results

Demographic, anthropometric, and metabolic characteristics of the whole study population are reported (Table 1). Thirty-six percent of subjects were men (*n* = 171). Eighty-two percent of the study population presented excessive body weight (*n* = 390) according to the BMI classification of the World Health Organization (BMI ≥ 25 kg/m^2^). Regarding the reference values, the metabolic profile of the whole population was characterized by increased blood levels of glucose and total cholesterol.

**Table 1:** Demographic, anthropometric, and metabolic characteristics as well as dietary intake of the whole study population (*n* = 474)

Variable	Average values
Age (years)	47.2 ± 14.1
Men/women	171/303
Anthropometric and clinical data	
Weight (kg)	81.6 ± 19.1
BMI (kg/m^2^)	30.1 ± 5.6
WC (cm)	95.7 ± 16.1
MAP (mmHg)	100.4 ± 16.2
Metabolic profile	
Glucose (mmol/L)	5.7 ± 1.7
Insulin (pmol/L)	66.7 ± 48.6
HOMA-IR index	2.44 ± 2.28
Total cholesterol (mmol/L)	5.3 ± 1.0
Triglycerides (mmol/L)	1.4 ± 0.8
TyG index	4.61 ± 0.32
Dietary intake	
Energy (Kcal/day)	2576 ± 759
Carbohydrates (g/day)	260.9 ± 96.8
Protein (g/day)	103.8 ± 28.3
Fat (g/day)	115.3 ± 36.4

Continuous variables are represented as means ± standard deviations. Men and women are number of cases. *BMI* body mass index, *WC* waist circumference, *MAP* mean arterial pressure, *HOMA-IR index* homeostatic model assessment-insulin resistance index, *TyG index* triglyceride-glucose index. Dietary intake was available from 247 subjects

The first screening revealed that 61 CpG sites at genes participating in the olfactory transduction pathway correlated with the BMI (*p* < 0.05). Out of these, 35 CpGs showed best correlations with BMI based on stricter FDR values (*p* < 0.0001). After performing linear regression tests adjusted by sex and age, 15 CpG sites still remained statistically significant. These CpG sites comprised cg19302979 (*SLC8A1*), cg02874396 (*OR4D2*), cg10610428 (*ANO2*), cg12498094 (*SLC8A1*), cg07736155 (*PDE2A*), cg17283169 (*CALML3*), cg02849894 (*GNG7*), cg15102821 (*CALML6*), cg16401207 (*PRKG1*), cg00467296 (*OR51A7*), cg24609819 (*PRKG1*), cg13801347 (*CAMK2D*), cg15819352 (*CALML6*), cg13441213 (*OR2T34*), and cg18482656 (*OR2Y1*).

**Fig. 1.**
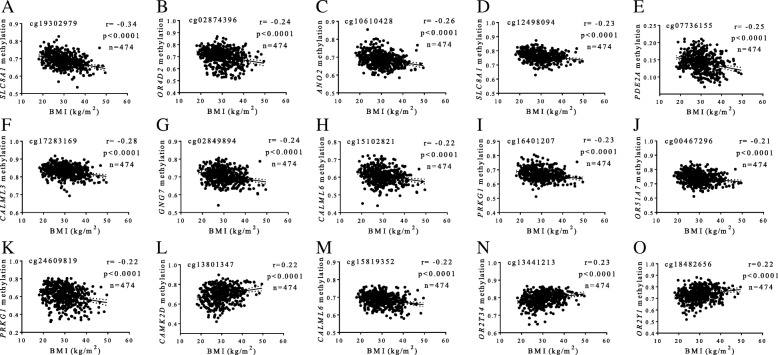
Associations between methylation levels (beta values) at olfactory pathway genes and BMI values. **a** cg19302979, *SLC8A1* (**b**) cg02874396, *OR4D2* (**c**) cg10610428, *ANO2* (**d**) cg12498094, *SLC8A1* (**e**) cg07736155, *PDE2A* (**f**) cg17283169, *CALML3* (**g**) cg02849894, *GNG7* (**h**) cg15102821, *CALML6* (**i**) cg16401207, *PRKG1* (**j**) cg00467296, *OR51A7* (**k**) cg24609819, *PRKG1* (**l**) cg13801347, *CAMK2D* (**m**) cg15819352, *CALML6* (**n**) cg13441213, and *OR2T34* (**o**) cg18482656, *OR2Y1*

**Fig. 2:**
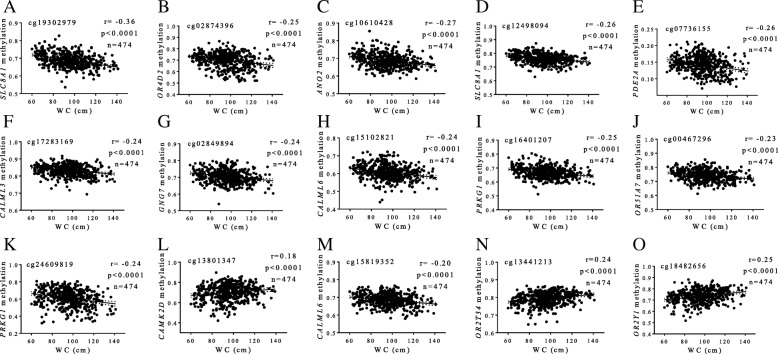
Associations between methylation levels (beta values) at olfactory pathway genes and WC values. **a** cg19302979, *SLC8A1* (**b**) cg02874396, *OR4D2* (**c**) cg10610428, *ANO2* (**d**) cg12498094, *SLC8A1* (**e**) cg07736155, *PDE2A* (**f**) cg17283169, *CALML3* (**g**) cg02849894, *GNG7* (**h**) cg15102821, *CALML6* (**i**) cg16401207, *PRKG1* (**j**) cg00467296, *OR51A7* (**k**) cg24609819, *PRKG1* (**l**) cg13801347, *CAMK2D* (**m**) cg15819352, *CALML6* (**n**) cg13441213, and *OR2T34* (**o**) cg18482656, *OR2Y1*

Interestingly, most of the BMI-associated CpGs were mapped to coding regions (*n* = 7) or terminal sequences (*n* = 2), and only six were located on gene promoters (Table 2). Together, methylation levels at these 15 CpG sites accounted for 22% of the variability in BMI (*r*^2^ = 0.219, *p* < 0.001). Illustrative correlations of each CpG site are reported (Fig. 1). Moreover, methylation signatures at the aforementioned 15 CpGs also correlated with WC values in a similar way (Fig. 2), explaining about 20% of variation in WC (*r*^2^ = 0.204, *p* < 0.001). No statistically significant relationships between methylation status at olfactory transducing genes with blood levels of metabolic markers and blood pressure measurements were found.

**Table 2:** Genomic and statistical data of CpG sites at olfactory pathway genes putatively associated with BMI

CpG_ID^1^	Illumina_ID	Gene name	Gene symbol	CHR position^2^	Genomic region	*p* value	FDR	B	*r* ^2^
1	cg19302979	Solute carrier family 8 member A1	*SLC8A1*	2: 40436843	Body	4.0 × 10^−12^	3.2 × 10^−09^	15.80	0.12
2	cg02874396	Olfactory receptor family 4 subfamily D member 2	*OR4D2*	17: 56245848	TSS1500	1.3 × 10^−10^	4.4 × 10^−08^	12.42	0.06
3	cg10610428	Anoctamin 2	*ANO2*	12: 5884295	Body	1.5 × 10^−09^	2.7 × 10^−07^	10.01	0.07
4	cg12498094	Solute carrier family 8 member A1	*SLC8A1*	2: 40356782	Body	1.4 × 10^−08^	1.4 × 10^− 06^	7.81	0.05
5	cg07736155	Phosphodiesterase 2A	*PDE2A*	11: 72354100	TSS1500	1.7 × 10^−08^	1.6 × 10^− 06^	7.65	0.07
6	cg17283169	Calmodulin-like 3	*CALML3*	10: 5567524	3′UTR	2.1 × 10^−08^	2.0 × 10^− 06^	7.42	0.08
7	cg02849894	G protein subunit gamma 7	*GNG7*	19: 2608971	5′UTR	4.5 × 10^−08^	3.4 × 10^−06^	6.68	0.06
8	cg15102821	Calmodulin-like 6	*CALML6*	1: 1844801	TSS1500	1.4 × 10^−07^	7.6 × 10^− 06^	5.61	0.05
9	cg16401207	Protein kinase, cGMP-dependent, type I	*PRKG1*	10: 53182118	Body	1.5 × 10^−07^	8.0 × 10^−06^	5.54	0.05
10	cg00467296	Olfactory receptor family 51 subfamily A member 7	*OR51A7*	11: 4928760	1stExon	1.9 × 10^−07^	9.7 × 10^−06^	5.28	0.04
11	cg24609819	Protein kinase, cGMP-dependent, type I	*PRKG1*	10: 52840377	Body	3.8 × 10^−07^	1.6 × 10^−05^	4.62	0.05
12	cg13801347	Calcium/calmodulin dependent protein kinase II delta	*CAMK2D*	4: 114460056	Body	1.7 × 10^−06^	4.7 × 10^−05^	3.14	0.05
13	cg15819352	Calmodulin-like 6	*CALML6*	1: 1845940	TSS1500	2.1 × 10^−06^	5.4 × 10^−05^	2.96	0.05
14	cg13441213	Olfactory receptor family 2 subfamily T member 34	*OR2T34*	1: 248738754	TSS1500	3.0 × 10^−06^	6.9 × 10^−05^	2.62	0.05
15	cg18482656	Olfactory receptor family 2 subfamily Y member 1	*OR2Y1*	5: 180167965	TSS1500	3.6 × 10^−06^	7.9E^−05^	2.45	0.05

Data are sorted by FDR values

*BMI* body mass index, *CHR* chromosome, *FDR* false discovery rate, *B* LIMMA B-statistic from LIMMA

^1^Studied CpG identifier

^2^CpG locations were mapped using GRCh37 version of the genome from Ensembl platform

**Fig. 3:**
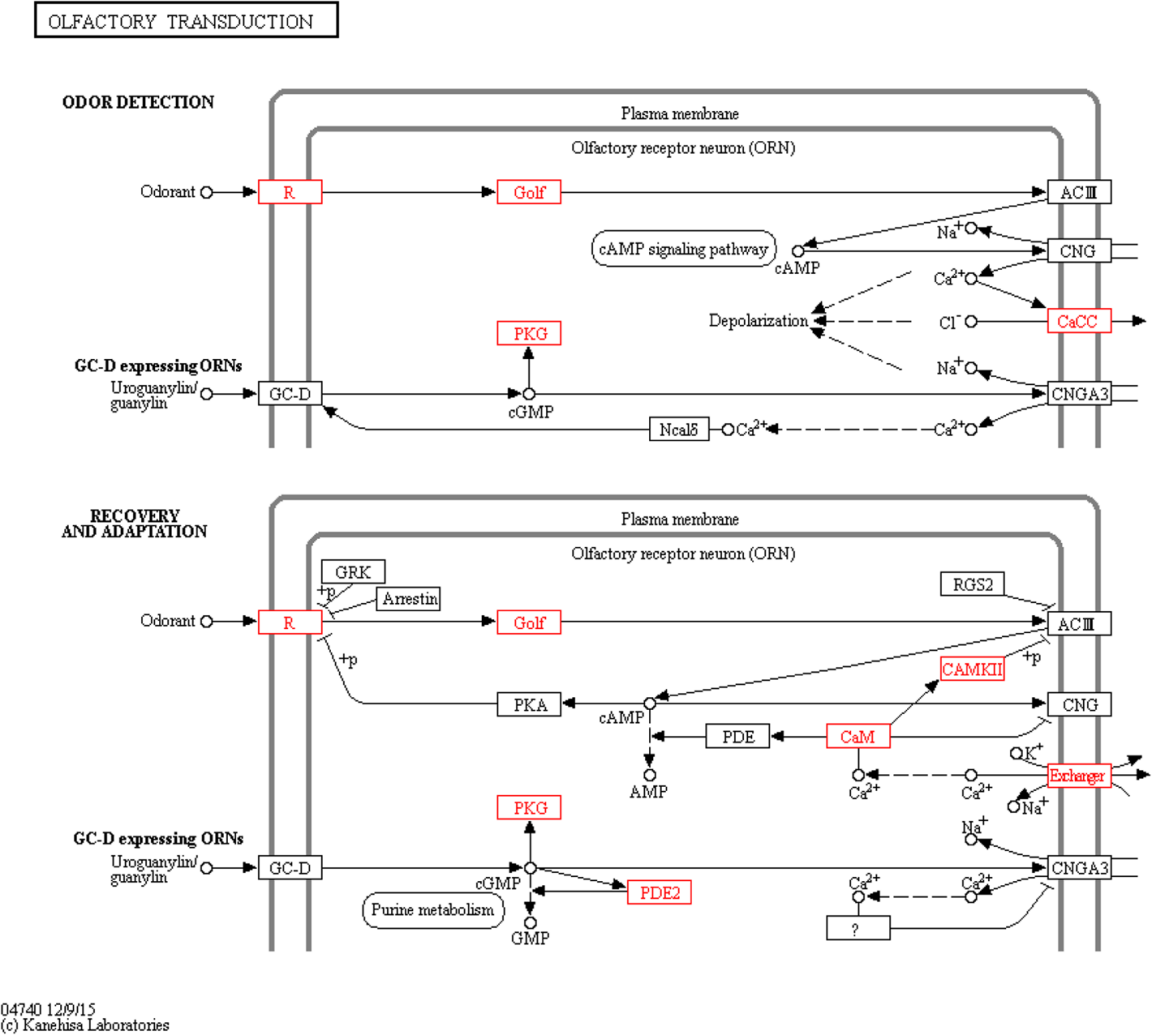
Pathway mapping of BMI-associated genes within the olfactory transduction network (red boxes). The following genes were computed: *SLC8A1*, *OR4D2*, *ANO2*, *PDE2A*, *CALML3*, *GNG7*, *PRKG1*, *OR51A7*, *CAMK2D*, *CALML6*, *OR2T34*, and *OR2Y1*. Figure taken from KEGG reference database (map04740). Pathway enrichment analyses, based on pathDIP (*p* = 1.94 × 10^−10^)

Pathway mapping of the BMI-associated genes within the olfactory transduction cascade is shown (Fig. 3). Notably, pathway enrichment analysis revealed a significant contribution (*p* = 1.94 × 10^−10^) of these genes to the regulation of the olfactory transduction network, which were involved in odor detection and signal processing in the nervous system (Fig. 3). These genes included the olfactory receptors *OR4D2*, *OR51A7*, *OR2T34*, and *OR2Y1* and several downstream effectors, such as *SLC8A1*, *ANO2*, *PDE2A*, *CALML3*, *GNG7*, *CALML6*, *PRKG1*, and *CAMK2D*.

**Fig. 4:**
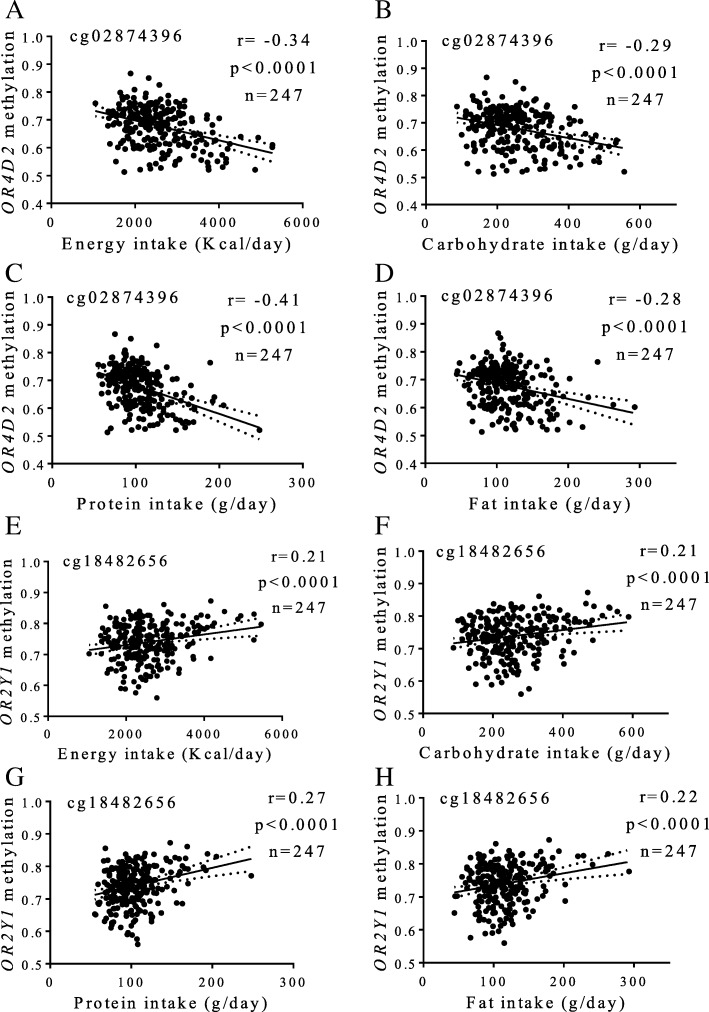
Associations between methylation levels (beta values) at olfactory receptors and dietary intakes. **a**–**d** cg02874396, *OR4D2* (**e**–**h**) cg18482656, *OR2Y1*

Furthermore, potential associations between olfactory receptor gene methylation status and dietary intakes were screened in a subsample of the MENA cohort (Fig. 4). Notably, methylation at cg02874396 (*OR4D2*) and cg18482656 (*OR2Y1*) strongly correlated with daily intakes of total energy (*p* < 0.0001), carbohydrates (*p* < 0.0001), protein (*p* < 0.0001), and fat (*p* < 0.0001).

## Discussion

Olfaction is considered an important sensorial factor influencing feeding behaviors through modulating food palatability and appetite [10, 13]. Thus, olfactory disruptions at the phenotypic and molecular levels may affect food intake and, consequently, individual predisposition to weight gain and obesity [9]. The current research shows, apparently for the first time, associations between different methylation patterns at olfactory pathway genes and adiposity indicators (BMI and waist circumference), which were independent of age and sex. These genes encompassed olfactory receptors (*OR4D2*, *OR51A7*, *OR2T34*, *OR2Y1*) and downstream signaling molecules (*SLC8A1*, *ANO2*, *PDE2A*, *CALML3*, *GNG7*, *CALML6*, *PRKG1*, *CAMK2D*), which significantly regulated odor detection and signal transduction within the complete olfactory cascade, as revealed by pathway analyses. Moreover, *OR4D2* and *OR2Y1* gene methylation patterns strongly correlated with daily energy and macronutrient intakes. The fact that methylation levels at 13 CpG sites negatively correlated with BMI, and only 3 CpG sites inversely positively associated with BMI, apparently reveal gene-specific profiling of DNA methylation regarding olfactory methylation status and obesity. These findings may contribute to elucidating novel relationships between olfactory system epigenetics, food consumption, and body weight homeostasis.

Odorant signal transduction is initiated when volatile odorants (including those emanating from food) interact with specific olfactory receptors in the nasal olfactory epithelium, leading to the initial perception of smell in the brain [44]. It has been reported that olfactory receptors are also expressed in non-chemosensory tissues, where they perform multiple physiological and metabolic functions [45]. The results found in this research are consistent with the role of olfactory perception in regulating food intake and energetic balance, as reported elsewhere [13, 14, 16]. Interestingly, genome-wide association analyses detected copy number variations in olfactory receptor genes that were associated with early-onset extreme obesity in humans [23]. Also, predicted damaging missense variants in olfactory receptor and protocadherin beta cluster genes were co-localized in subjects with extreme obesity [24]. Similarly, olfactory receptor gene polymorphisms showed evidence of an association with adiposity levels and some eating behaviors, including cognitive dietary restraint, susceptibility to hunger, and eating disinhibition [25].

Regarding odor-evoked transducers, PRKG1, a cGMP-dependent protein kinase, involved in foraging behavior, food acquisition, and energy balance, was located within a variably methylated region associated with BMI in a human cohort [46]. Until now, the specific roles of *ANO2*, *CAMK2D*, *SLC8A1*, *PDE2A*, *CALML3*, *GNG7*, and *CALML6* genes in olfactory-related dietary patterns and obesity in humans have not been apparently explored; therefore, further investigation in these research areas is warranted.

Potential relationships between eating patterns, olfactory function, and obesity have also been phenotypically studied. In this context, consumers of a Western-style diet (rich in saturated fat and added sugar) presented poorer odor identification ability, worse fat discrimination, and hedonic differences in taste and flavor perception relative to people who consumed a healthier diet [47]. Of note, an increasing BMI has been associated with a decrease in olfactory sensitivity [48]. High BMI was also related to subjective olfactory dysfunction in obese patients [49]. In addition, an impaired olfactory capacity has been reported in obese subjects compared to normal-weight controls [50]. Furthermore, an increase in visceral fat content was associated with a decrease in olfactory function [51].

To the best of our knowledge, this is a pioneer study exploring the role of epigenetics of olfaction in obesity. The strengths of this investigation include the screening of the whole olfactory transduction pathway and the relative large number of DNA samples analyzed. Moreover, methylation-related statistical analyses were adjusted for potential confounding factors such as sex, age, study cohorts, methylation chips, and cell composition variability as well as with corrections for multiple comparisons within the experiments performed in this study. Instead, the limitations of this study encompass those inherent to retrospective association studies including the inability to explain cause-effect relationships, the scientific caution concerning the reproducibility of the findings in other administrative settings having their own peculiar biases, and the difficulties in distinguishing between factors related to increased or decreased risk of developing the disease and those associated with the course of the illness [52]. Other drawbacks of this study comprise the exclusion of expression assays (RNA samples were not available) and the lack of tests evaluating olfactory function. Also, given the number of subjects analyzed and the type of study (association) type I and type II bias cannot be completely excluded despite the statistical settings. Likewise, some methylation relevant data related to obesity could have not been taken into account due to the use of rigorous FDR thresholds in the outcome-related analyses.

Another issue of this study could be the fact that DNA methylation levels were measured in circulating white blood cells instead of olfactory tissues. Interestingly, the expression of some olfactory receptors has been detected in different blood cells, including *OR51A7* in leukocytes [53]. Also, our results are in accordance with a previous investigation showing associations between methylation signatures at sweet taste transducing genes, obesity, and carbohydrate intake [26]. Additionally, some studies support that methylation marks in blood cells can mirror those found in other samples, including oral mucosa [54] and subcutaneous adipose tissue [55], suggesting the possible use of the methylome in leukocytes for disease-risk prediction and therapeutic purposes.

In recent years, the implication of different epigenetic processes in the development of obesity is being extensively investigated [56]. Especially, altered DNA methylation patterns can trigger changes in gene expression associated with deregulations of energy homeostasis and weight gain predisposition [7]. In this manner, genes integrating the olfactory pathway are susceptible to covalent epigenetic modifications that might lead to odor perception dysfunction. Establishing an epigenetic basis for olfactory function may help to understand, at least in part, relationships between olfaction capacity, food consumption, and body weight regulation. In turn, this knowledge may contribute to identify epigenetic biomarkers to predict the risk of developing excessive adiposity and associated comorbidities, as well as implement epigenome-based dietary strategies for prevention, prognosis, and treatment of obesity within the era of precision nutrition [57].

## Conclusions

The results of this investigation suggest novel relationships between olfactory pathway gene methylation signatures, obesity indices, and dietary intakes.
